# Siamese anchor-free object tracking with multiscale spatial attentions

**DOI:** 10.1038/s41598-021-02095-4

**Published:** 2021-11-25

**Authors:** Jianming Zhang, Benben Huang, Zi Ye, Li-Dan Kuang, Xin Ning

**Affiliations:** 1 School of Computer and Communication Engineering, Changsha University of Science and Technology, Changsha, 410114 China; 2 Hunan Provincial Key Laboratory of Intelligent Processing of Big Data On Transportation, Changsha University of Science and Technology, Changsha, 410114 China; 3 Institute of Semiconductors, Chinese Academy of Sciences, Beijing, 100083 China

**Keywords:** Engineering, Electrical and electronic engineering, Mathematics and computing, Computer science

## Abstract

Recently, object trackers based on Siamese networks have attracted considerable attentions due to their remarkable tracking performance and widespread application. Especially, the anchor-based methods exploit the region proposal subnetwork to get accurate prediction of a target and make great performance improvement. However, those trackers cannot capture the spatial information very well and the pre-defined anchors will hinder robustness. To solve these problems, we propose a Siamese-based anchor-free object tracking algorithm with multiscale spatial attentions in this paper. Firstly, we take ResNet-50 as the backbone network to generate multiscale features of both template patch and search regions. Secondly, we propose the spatial attention extraction (SAE) block to capture the spatial information among all positions in the template and search region feature maps. Thirdly, we put these features into the SAE block to get the multiscale spatial attentions. Finally, an anchor-free classification and regression subnetwork is used for predicting the location of the target. Unlike anchor-based methods, our tracker directly predicts the target position without predefined parameters. Extensive experiments with state-of-the-art trackers are carried out on four challenging visual object tracking benchmarks: OTB100, UAV123, VOT2016 and GOT-10k. Those experimental results confirm the effectiveness of our proposed tracker.

## Introduction

Object tracking, aiming to predict the position of a target given in the initial frame of a video sequence in each subsequent frame, is a fundamental yet challenging task in the field of computer vision. Object tracking has received much attention, because of the wide range of application scenarios, such as video surveillance, robotic vision navigation medical diagnosis and augmented reality. Although much remarkable progress has been achieved in recent years, it still faces multiple challenges mainly from the two aspects: (1) the outside environment: background clutter, illumination variation, low resolution, full occlusion, etc.; (2) the inside target itself: rotation, scale variation, deformation, etc.

Recently, visual object tracking algorithms have been receiving continuous attentions, which can be roughly divided into two branches: one is based upon correlation filter, the other is based upon deep learning. The correlation filter-based (CF) trackers train a regressor of a target given in the initial frame of a video, and use this regressor with Fourier transforming to calculate the location of the target in the candidate region. Those CF-based trackers can track the object online, and update the parameters of filters during this process efficiently. KCF^1^ introduces kernel trick into correlation filter, which maps the ridge regression in linear space to a high-dimensional nonlinear feature space, to get better performance. Hand-crafted features are used in those works^2–6^ to get more comprehensive appearance representations. Those methods^7–10^ use multiscale features to improve tracking accuracy. Besides several methods^11–13^ combine both deep features and hand-crafted features to get better performance. As time goes by, the convolutional neural networks (CNN)-based methods have made great performance in many domains, such as object detection, image processing^14–18^. The CNN-based object tracking methods have achieved great success during in recent years, which mainly have two categories. The one is widely-used Siamese-based trackers^19–21^ which usually stores the appearance information of the initial target as an explicit template. The other intends to store the appearance information as the fine-tuned parameters into the neural network^22^.

Recently, Siamese-based methods, the mainstream branch of deep learning method, have become popular due to their considerable performance. Siamese Fully-Convolutional (SiamFC)^19^ first introduces Siamese network into visual object tracking, which transforms the tracking problem into similarity calculation problem between target and search region. SiamFC constructs a lightweight Siamese network to extract target and search area features respectively. The target bounding box is determined according to the maximum position of the response map. After offline training, the parameters of the network won’t be updated during the tracking process. Siamese region proposal network (SiamRPN)^20^ proposes a region proposal network (RPN) after Siamese feature extraction, which removes the time-consuming scale pyramid and improves the speed and accuracy of FC-based trackers^19,23^. The RPN module turns the similarity learning problem to a classification and regression problem. After that, many advanced trackers, like Distractor-aware Siamese Region Proposal Networks (DaSiamRPN)^21^, SiamMask^24^ and SiamRPN++^25^, improve SiamRPN. The above RPN-based algorithms obtain accurate target bounding boxes by designing multiscale anchor boxes, which not only seriously affect the robustness but also increase the interference of human factors.

**Figure 1:**
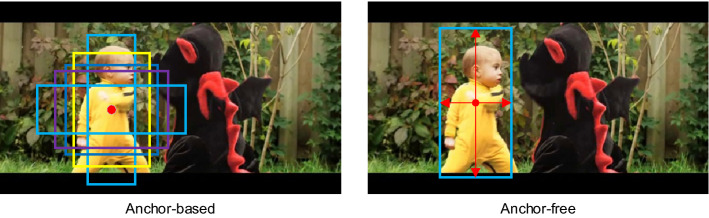
The left side is the anchor-based method which uses the fixed different ratio aspects anchors to locate the location of an object, and the right side is the anchor-free method that directly estimate the bounding box.

In our work, we propose a Siamese-based anchor-free algorithm with multiscale spatial attentions to solve the above problems. Our proposed framework consists of three following subnetworks. First, we use the ResNet-50^26^ as backbone of our framework to extract the multilevel features for both template and search regions. Second, we design a spatial attention extraction (SAE) block to catch the long-range dependencies between the features extracted from the different layers of ResNet-50. As shown in Fig. 1, the anchor-based trackers usually determine the bounding boxes with the different ratio anchors. Third, inspired by those state-of-the-art anchor-free detectors^27–29^, we design a classification-regression subnetwork to track object without the pre-defined operations or parameters. We directly predict the foreground and background score of the target, and regress a 4-channel vector representing the distance from the corresponding position of each pixel in the response map to the four sides of the ground-truth boxes.

Our main contributions of this work are as follows:

1. We propose a Siamese anchor-free network with multiscale spatial attentions for visual object tracking, and use the modified ResNet-50 as backbone to extract multiscale features from both template and search region.
2. We design a SAE block to generate the spatial information among all positions in the template and search region feature maps. We then put the multiscale features into the SAE block to generate multiscale spatial attentions. The multiscale spatial information can help our model distinguish between foreground and background more precisely.
3. We use an anchor-free classification and regression subnetwork with the multiscale spatial attention to predict the template label and calculate the prediction bounding boxes. Without the pre-defined parameters, our tracker is more flexible and can regress the bounding box more accurately.
4. The whole network of our tracker is trained offline on five datasets, including COCO^30^, Imagnet^31^, YouTube-BoundingBoxes^32^, YouTube-VOS^33^, GOT-10k^34^, and achieves considerable results on the four mainstream challenging visual object tracking benchmarks: OTB100^35^, UAV123^36^, VOT2016^37^ and GOT-10k^34^. The success and the precision scores are 0.673 and 0.900 on the OTB100 dataset. On UAV123, the success and the precision scores can achieve 0.595 and 0.790, respectively. The accuracy, robustness and expected average overlap (EAO) score are 0.618, 0.172 and 0.448 on the VOT2016 dataset. On the GOT-10k dataset, the AO, ${SR}_{0.50}$ and ${SR}_{0.75}$ are 0.549, 0.660 and 0.377 respectively. The code and results are available at: https://github.com/csust7zhangjm/Siamese-Anchor-free-Object-Tracking-with-Multiscale-Spatial-Attentions.

## Related work

Object tracking, a Basic yet challenging task in the field of computer vision, attracts increasing attention due to its balanced efficiency and accuracy in recent years. In this section, we provide a comprehensive review of the existing methods relevant to our work in three areas: Siamese-based object trackers, attention mechanisms and anchor-free object detectors.

### Siamese-based object trackers

The core of Siamese network is to construct fully convolutional network, which contains two weights-sharing branches. They are used to extract and save the features of the template patches and the search region, respectively. Siamese instance search tracker (SINT)^38^, the early Siamese tracker, divides the network into query stream and search stream based on similarity learning. The matching function in SINT is used to find the most suitable candidate region, but the speed is slow, just 2 frames per second (fps). SiamFC^19^ transforms the target tracking problem into similarity learning problem. By constructing lightweight Siamese network structure, the target features and search region features are extracted respectively, and the cross-correlation operations are carried out to combine those feature maps. SiamRPN^20^ introduces the RPN into Siamese network, and transforms the similarity calculation problem of SiamFC into the classification and regression problem. Because RPN module does not need the scale pyramid of SiamFC, SiamRPN shows the speed and the precision improvements compared to SiamFC. SiamMask^24^ uses the mask segmentation method to obtain the bounding box and mask at the same time. SiamRPN++^25^ extracts multi-level features by using ResNet-50 as backbone. The deeper and wider Siamese networks (SiamDW)^39^ designs the cropping-inside residual units to build deeper and wider algorithms to improve tracking performance. Although these optimizations make tracking better, the pre-defined anchor boxes not only lead ambiguous similarity score that seriously affects the robustness but also increase the interference of human factors.

### Anchor-free mechanisms

Due to their simple architectures but superior performance, anchor-free detectors have attracted wide attention in object detection recently. Different from the anchor-based approaches, anchor-free methods calculate the position of the target directly. You only look once (Yolov1)^40^ divides the image into a square gird, and predicts the location and the label of image on each grid unit. Unitbox^27^ introduces an Intersection over Union (IoU) loss to train the four boundary positions as a whole unit. FCOS^28^ regards each pixel in the ground-truth bounding box as positives, and predicts the labels of all pixels and regresses the distance from the corresponding position of each pixel to the border of the bounding box. Inspired by those anchor-free detectors, we introduce the anchor-free mechanism into our framework. There are several anchor-free trackers^41,42^ recently, which introduce some special methods to enhance trackers, like feature alignment or quality assessment. Different from them, our tracker takes the anchor-free framework with our own SAE block to track object.

### Attention mechanisms

Attention mechanisms can catch long-range dependencies and have been used in many fields including image classification, image segmentation and object tracking. SENet^43^ proposes a Squeeze-and-Excitation (SE) block to rescale the different channels to build interdependencies between channels. Convolutional Block Attention Module (CBAM)^44^ proposes an efficient module to exploit both spatial and channel attention, which improves the performance compared to SENet. Non-Local Networks (NLNet)^45^ introduces a NL operation to get the long-range dependencies, and can be easily inserted into any structure. Inserting attention mechanisms into Siamese network is not a new concept. SA-Siam^23^ is a twofold Siamese object tracking algorithm consisting of an appearance branch and a semantic branch. In the semantic branch, SA-Siam proposed a channel attention module to calculate the channel-wise attention. There are three different kinds of attention mechanisms using in Residual Attentional Siamese Network (RASNet)^46^, including general attention, residual attention, and channel attention. In our work, we design a SAE block after Siamese network, which aims to better explore the potentials of different layers in Siamese network.

## Methods description

**Figure 2:**
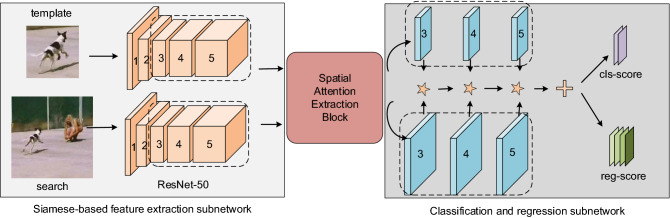
The overall of our Siamese anchor-free object tracking with multiscale spatial attention tracker, which consists of three modules: the Siamese-based subnetwork, the multiscale SAE block and the classification and regression subnetwork. The Siamese-based subnetwork (left side) utilizes the ResNet-50 as backbone to extract the feature of the last three stages for both the template branch and the search area branch. The backbone of these two branches shares the same structure. Those features are modified by the SAE block. The classification and regression subnetwork (right side), which takes the multiscale spatial attention features as input to predict the position of the target in search region. $\star$ denotes the depth-wise convolution operation. $+$ denotes the channel-wise addition operation.

In this section, we describe the details of our model. As we can see in Fig. 2, the overall framework manly consists of three modules: the Siamese-based subnetwork, the multiscale SAE block and the classification and regression subnetwork. The Siamese-based subnetwork is used for extract the features of the template branch and the search region branch with an offline manner. The proposed SAE block captures long-range dependency among all positions effectively. The classification-regression subnetwork is a multi-level anchor-free structure, and have classification and regression branches. The classification branch is responsible for predicting the foreground–background label on each pixel of the feature map. The regression branch is used for bounding box prediction on the corresponding position of each point of the feature map.

### Siamese-based feature extraction subnetwork

SiamFC^19^ introduces the Siamese network into visual object tracking field, which views the visual object tracking as a similarity calculating problem. And the whole framework is trained offline, and consists of two branches which share the same parameters in CNN. One branch is the template branch that takes the target patch (denotes as $z$) given in the first frame as input. The other is the search branch taking the search region as input (denotes as $x$). Modern deep convolutional neural networks^25,39^ have proven to be robust and accuracy as in object tracking. In our tracker, we take ResNet-50^26^ as backbone for feature extraction. The outputs of the two branches are regard as $\varphi (x)$ and $\varphi (z)$ respectively. To better utilize the detailed spatial information for prediction, we remove the down-sampling operations from the last two bottleneck layers. We replace the $3\times 3$ convolutions in the last two bottleneck layers of ResNet-50 by the dilated convolution operation^47^ with the strides are modified to 1 and the dilation rates are set to $\left(a,b\right)\in \{\left(\mathrm{2,2}\right),\left(\mathrm{4,4}\right)\}$, separately.

**Figure 3:**
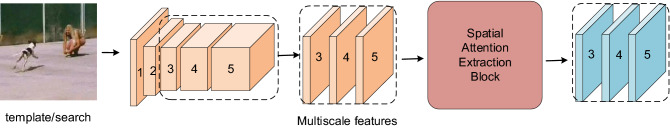
Multiscale spatial attention extraction process of template or search region images.

Features from different layers can provide different effects for tracking. The features from earlier layers containing low-level information are indispensable for localization, while features from latter layers having abstract semantic information are more essential for discrimination. Inspired by those methods^25,39^, we extract features from the last three residual block of ResNet-50, as shown in the left side of Fig. 3. We regard the outputs of the last three layers as. ${\varphi }^{3}(x),{ \varphi }^{4}(x),{ \varphi }^{5}(x)$ and ${\varphi }^{3}(z),{ \varphi }^{4}(z),{ \varphi }^{5}(z)$, respectively:

1 $$\begin{array}{c}\genfrac{}{}{0pt}{}{\varphi \left(x\right) \, = \, Cat\left({\varphi }^{3}\left(x\right),{\varphi }^{4}\left(x\right),{\varphi }^{5}\left(x\right)\right),}{\varphi \left(z\right) \, = \, Cat\left({\varphi }^{3}\left(z\right),{\varphi }^{4}\left(z\right),{\varphi }^{5}\left(z\right)\right),}\end{array}$$

where $\varphi \left(\cdot \right)$ denotes the features extraction operation of the template patch and the search region. After the feature extraction operation, we use three $1\times 1$ convolution layers ($conv1\times 1$) to reduce the channels of ${\varphi }^{i}(l)(l=x,\mathrm{z}; i=\mathrm{3,4},5)$ to 256, respectively. Therefore, $\varphi (x)$ and $\varphi (z)$ include $3\times 256$ channels, simultaneously.

### Multiscale spatial attention extraction subnetwork

#### Spatial attention extraction block

**Figure 4:**
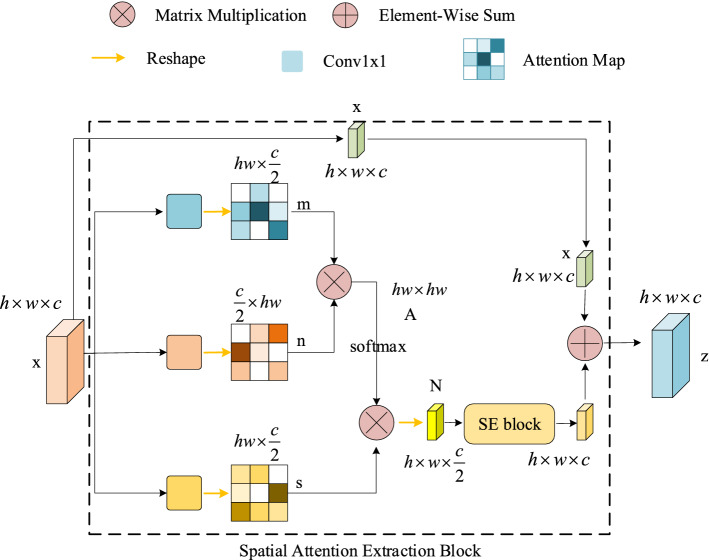
The proposed SAE block, which consists of three blocks: a NL context modeling block, a SE transforming block and a residual block. It takes template features and search region features as inputs, and calculates the spatial attentions of both branches.

In order to accurately pinpoint the borders of the target, it is important to use global contextual information. The Squeeze-and-excitation networks (SENet)^43^ can capture the channel-wise independencies. The Non-local Neural Networks (NLNet)^45^ can effectively obtain the long-range dependencies through calculating the response map as a weighted sum of all location features in the input feature map. Inspired by the SE module and the NL module, we propose a SAE block. As shown in Fig. 4, the proposed module contains three blocks: a non-local (NL) context modeling block, a squeeze-excitation (SE) transforming block and a residual block. The proposed SAE block takes the feature maps of both target and search images computed from feature extracted network as input. Taking the target image for example. We assume $x$ is the input features of the SAE block with the shapes of $h\times w\times c$. In non-local context modeling block, two $conv1\times 1$ are applied to reshape the input features to $m, n$ respectively, where $m\in {\mathbb{R}}^{N\times {c}^{\mathrm{^{\prime}}}},$ $n\in {\mathbb{R}}^{{c}^{\mathrm{^{\prime}}}\times N}$ and ${c}^{\mathrm{^{\prime}}}=0.5c, N=h\times w.$ The attention of the NL block representing the relationship between different pixels on the feature map can be generated via matrix multiplication and row-wise softmax operations as:

2 $$\begin{array}{c}A={softmax}_{row}\left(mn\right)\in {\mathbb{R}}^{N\times N}.\end{array}$$

At the same time, the $conv1\times 1$ reshape $x$ to $\mathrm{s}\in {\mathbb{R}}^{N\times {c}^{\mathrm{^{\prime}}}}$. The NL context attention features $N$ are generated:

3 $$\begin{array}{c}N=r\left(As\right)\in {\mathbb{R}}^{h\times w\times {c}^{\mathrm{^{\prime}}}},\end{array}$$

where $r(\cdot )$ is a reshape operation to make the feature size back to $h\times w\times {c}^{\mathrm{^{\prime}}}$. We then put the NL context attention features to the SE transforming block. The SE block contains one $conv1\times 1$, one batch normalization (BN), one ReLU and one $conv1\times 1$. After modifying by the SE transforming block, we can aggregate the spatial attentional features to the feature of each position with adding a residual module $x$ as:

4 $$\begin{array}{c}z=x+\varrho \left(N\right),\end{array}$$

where $\varrho \left(\cdot \right)=conv1\times 1(ReLU(BN(conv1\times 1(\cdot ))))$, which is the SE transforming operation to generate the channel-wise dependencies. Therefore, the complete calculation formula of the SAE block can be defined as:

5 $$\begin{array}{c}z=x+\varrho \left(r\left({softmax}_{row}\left(mn\right)s\right)\right)\in {\mathbb{R}}^{h\times w\times \mathrm{c}}.\end{array}$$

#### Multiscale spatial attention in Siamese network

In our work, we input the features of the last three layers of ResNet-50 of both template and search feature map into the SAE block. As shown in the right side of Fig. 3, we can get two multiscale spatial attention features for template and search region respectively, which help our tracker encode more global context information, defined as $g(\varphi \left(x\right))$ and $g(\varphi \left(z\right))$ respectively:

6 $$\begin{array}{c}g\left(\varphi \left(l\right)\right)=Cat\left(g\left({\varphi }^{3}\left(l\right)\right), g\left({\varphi }^{4}\left(l\right)\right),g\left({\varphi }^{5}\left(l\right)\right)\right),\end{array}$$

here $g(\cdot )$ is the whole spatial attention extraction operation, $l=x,z$.

### Classification and regression subnetwork

For every pixel $(i,j)$ in the feature map can be found a response region $\left(x,y\right)$ in the search patch. The anchor-based methods consider the corresponding position on the search area as the center of multi-scale anchor boxes, and predicts the classification score and regress the borders with taking the anchor boxes as reference. In contrast, our tracker classifies the target image patch and regresses the corresponding bounding box at each location directly.

Without anchor boxes, the classification score of each pixel reflects the reliability whether the target is in the corresponding position directly. As shown in Fig. 2, the subnetwork consists of two branches: a classification branch, and a regression branch. Each branch takes the multi-level spatial attention features as input. We modify and put the $g(\varphi \left(x\right))$ and $g(\varphi \left(z\right))$ to the corresponding module into the classification branch and regression branch, respectively: ${[g\left(\varphi \left(x\right)\right)]}_{cls}$, ${[g\left(\varphi \left(x\right)\right)]}_{reg}$ and ${[g\left(\varphi \left(z\right)\right)]}_{cls}$, ${[g\left(\varphi \left(z\right)\right)]}_{reg}$. We use a depth-wise convolution layer to generate the feature maps. Thus, we can get a classification map ${p}_{h\times w\times 2}^{cls}$, and a regression map ${p}_{h\times w\times 4}^{reg}$, denoted as:

7 $$\begin{array}{l}\genfrac{}{}{0pt}{}{{p}_{h\times w\times 2}^{cls}\,=\,{\left[g\left(\varphi \left(x\right)\right)\right]}_{cls}\star {\left[g\left(\varphi \left(z\right)\right)\right]}_{cls},}{{p}_{h\times w\times 4}^{reg}\,=\,{\left[g\left(\varphi \left(x\right)\right)\right]}_{reg}\star {\left[g\left(\varphi \left(z\right)\right)\right]}_{reg},}\end{array}$$

where $h$ and $w$ represent the width and the height of those feature maps, respectively. $\star$ denotes the depth-wise convolution operation. Each pixel in ${p}_{h\times w\times 2}^{cls}$ is a 2-channel vector representing the positive and negative activation scores at the corresponding position in the initial search region. Meanwhile every pixel in ${p}_{h\times w\times 4}^{reg}$ is a 4-channel vector, which denotes as $Q=(l,t,r,b)\in {\mathbb{R}}^{4}$ measuring the distance from the corresponding position to the borders of the prediction bounding box in the search area.

We put the multiscale spatial attentional features into the classification and regression branch respectively. Therefore, we can get three pairs of prediction feature maps. The final classification feature maps and regression feature maps can be respectively fused:

8 $$\begin{array}{c}\genfrac{}{}{0pt}{}{{C}_{all}\,=\,\sum_{l=3}^{5}{\alpha }_{l}*{p}_{h\times w\times 2}^{cls,l},}{{R}_{all}\,=\,\sum_{l=3}^{5}{\beta }_{l}*{p}_{h\times w\times 4}^{reg,l},}\end{array}$$

where ${\alpha }_{l}$ and ${\beta }_{l}$ are the weights for classification and regression, separately, and trained together with the network.

We make $B=\left({x}_{0},{y}_{0},{x}_{1},{y}_{1}\right)\in {\mathbb{R}}^{4}$ denote the left-top and right-bottom corners of the ground-truth box of the target. Each pixel $\left(i,j\right)$ in the final feature map can be considered as a positive label if the corresponding location $\left({x}_{i},{y}_{i}\right)$ falls within the ground-truth box $B.$ The distance from the coordinates $\left({x}_{i},{y}_{i}\right)$ of the positive point $\left(i,j\right)$ to the ground-truth box can be calculated as $\tilde{Q }=\left(\tilde{l },\tilde{t },\tilde{r },\tilde{b }\right)\in {\mathbb{R}}^{4}$:

9 $$\begin{array}{c}{\tilde{l}\,=\,{x}_{i}-{x}_{0},\,\tilde{t}\,=\,{y}_{i}-{y}_{0},\, \tilde{r}\,=\,{x}_{1}-{x}_{i},\,\tilde{b}\,=\,{y}_{1}-{y}_{i}.}\end{array}$$

With $Q=(l,t,r,b)$ and $\tilde{Q }=(\tilde{l },\tilde{t },\tilde{r },\tilde{b })$, the IoU between the prediction bounding box and the ground-truth bounding box of each positive pixel can be calculated.

To further optimize our model, we use a binary cross-entropy (BCE)^48^ loss and a IoU^27^ loss to train the classification and regression networks respectively. The loss in regression branch is defined as:

10 $$\begin{array}{l}{L}_{reg}=\sum_{\forall i,j}\frac{1}{\sum \mathcal{G}\left(\tilde{Q }\right)}\mathcal{G}\left(\tilde{Q }\right){L}_{IoU}\left(Q,\tilde{Q }\right).\end{array}$$

Inspired by GIoU^49^, we define ${L}_{IoU}\left(Q,\tilde{Q }\right)=1-IoU(Q,\tilde{Q })$, and $\mathcal{G}\left(\tilde{Q }\right)$ is an operation to judge whether $\left({x}_{i},{y}_{i}\right)$ is in the ground-truth box, defined by:

11 $$\begin{array}{l}\mathcal{G}\left(\tilde{Q }\right)\, = \,\left\{\begin{array}{l}1 \quad if\,\, {\tilde{Q }}^{k}>0, \quad k\, = \,\mathrm{0,1},\mathrm{2,3}\\ 0 \quad otherwise \end{array}\right..\end{array}$$

Therefore, the overall loss function is calculated as follows:

12 $$\begin{array}{c}L={{\lambda }_{1}L}_{cls}+{{\lambda }_{2}L}_{reg},\end{array}$$

where ${L}_{cls}$ and ${L}_{reg}$ represent the BCE loss function and the IoU loss function respectively, meanwhile ${\lambda }_{1}$ and ${\lambda }_{2}$ are the weights of those loss functions, which are set to 1 empirically in our implementation.

## Results and analysis

### Implementation details

Our tracker is implemented in python 3.7 with PyTorch 1.7.1 on 3 RTX2080ti. We use the modified ResNet-50 as backbone of our proposed tracker, and its weights are pre-trained on the ImageNet^31^. By following SiamFC^19^, the template patches with $127\times 127$ pixels and the search regions with $255\times 255$ pixels are used for both training and testing.

#### Training

Our entire network is trained with six lager datasets: COCO^30^, YouTube-BoundingBoxes^32^, GOT-10k^34^, ImageNet-VID^31^, YouTube-VOS^33^, ImageNet-DET^31^. We train our model with stochastic gradient descent (SGD) and set the minibatch to be 28 pairs. We train our model for 20 epochs, which takes 60 h to finish training. In the first 5 epochs, we use a warmup learning rate from 0.001 to 0.005. Meanwhile, an exponentially decayed from 0.005 to 0.00005 learning rate is used for the last 15 epochs. For the first 10 epochs, we only train the multiscale SAE block and the classification-regression subnetwork with the parameters of the Siamese-based subnetwork frozen. For the last 10 epochs, we train the whole network together.

#### Testing

We follow the same strategy as in SiamFC^19^ and SiamRPN^20^ to test our proposed tracker. Take the target in the first frame of a video as the template patch, and then match it in the subsequent video search sequence. We evaluate the performance of our proposed algorithm on four widely-used object tracking benchmark datasets, including OTB100^35^, UAV123^36^, VOT2016^37^ and GOT-10k^34^.

### Quantitative evaluation with state-of-the-art tracker

#### On OTB100

**Figure 5:**
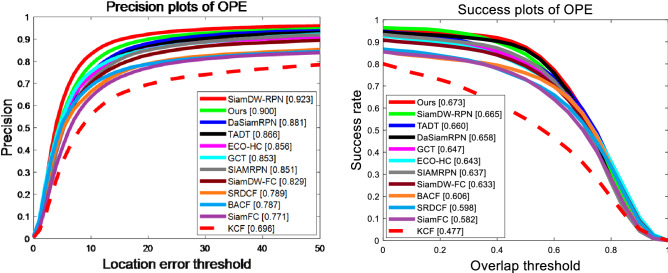
Precision and success plots of our tracker and 11 excellent trackers on OTB100.

The classical OTB100 benchmark dataset, contains one hundred videos, is widely used in evaluation for visual object tracking. OTB100 ranks trackers using area under curve (AUC) and precision (Prec.). We compare our algorithm with 11 advanced methods on the OTB100 dataset, including KCF^1^, SRDCF^3^, BACF^4^, ECO^12^, SiamFC^19^, SiamRPN^20^, DaSiamRPN^21^, SiamDW^39^, TADT^50^, GCT^51^. As can be seen in Fig. 5, the performance of our tracker is relatively excellent among those compared models. Although the precision score of our tracker ranks second blew SiamDW-RPN^39^ by 2.3% reached 0.900, the success rate of our tracker outperforms these trackers reached 0.673.

#### On UAV123

**Figure 6:**
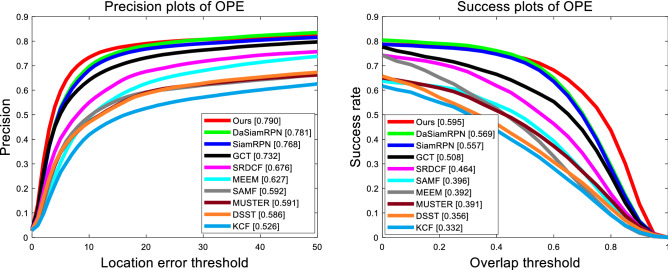
Precision and success plots of our tracker and 9 excellent trackers on UAV123.

The UAV123 benchmark dataset can be divided into three parts: the first 103 video sequences by UAV-stabilized cameras; the middle 12 video sequences by UAV-unstable cameras; the last 8 video sequences by UAV simulator. The evaluating indicators of UAV123 are the same as OTB100. The objects in UAV123 suffer from many challenges including large-scale variation, occlusions, and are small which make tracking tasks more difficult. We compare our algorithm with the recently-developed 9 methods, that is, KCF^1^, SAMF^2^, SRDCF^3^, SiamRPN^20^, DaSiamRPN^21^, GCT^51^, MEEM^52^, MUSTer^53^, DSST^54^ on this dataset for evaluation. As we can see in Fig. 6, our tracker achieves the considerable performance in both precision and success among these trackers. We achieve the precision of 0.790 and the success rate of 0.595, which both outperforms those classical anchor-based trackers (DaSiamRPN^21^ and SiamRPN^20^).

#### On VOT2016

The VOT2016 dataset is made of 60 videos with various challenges. The VOT2016 benchmark evaluates the overall performance of a tracker from three aspects: accuracy (A), robustness (R) and expected average overlap (EAO). Specially, the EAO is the combination of both R and A. The following advanced methods, including MCCT^9^, ECO^12^, SiamRPN^20^, DaSiamRPN^21^, SiamMask^24^, SiamRPN++^25^, SiamDW^39^, TADT^50^, ASRCF^55^ are put on VOT2016 for evolution. Table 1 shows the comparison at VOT2016. We achieve the top-3 performance among those compared trackers, which are 0.448 in EAO, 0.618 in accuracy and 0.172 in robustness. Especially in terms of robustness, our trackers run the first, better than the compared trackers, like SiamMask^24^, SiamRPN++^25^, DaSiamRPN^21^, which are 0.233, 0.177 and 0.224.

**Table 1:** Performance comparisons of our tracker with 9 excellent trackers on VOT2016.

Tracker	EAO $\uparrow$	A $\uparrow$	R $\downarrow$
MCCT	0.393	0.579	***0.186***
SiamRPN	0.337	0.578	0.312
SiamDW-RPN	0.376	0.574	0.266
DaSiamRPN	0.401	0.609	0.224
TADT	0.301	0.551	0.326
ECO	0.374	0.555	0.200
ASRCF	0.391	0.568	***0.186***
SiamMask	***0.442***	**0.670**	0.233
SiamRPN++	**0.478**	*0.637*	*0.177*
Ours	*0.448*	***0.618***	**0.172**

Bold, Italic and bold-italic fonts represent the top-3 trackers on each indicator. $\uparrow$ denotes the highest is the best, and $\downarrow$ denotes the lowest is the best.

#### On GOT-10k

**Figure 7:**
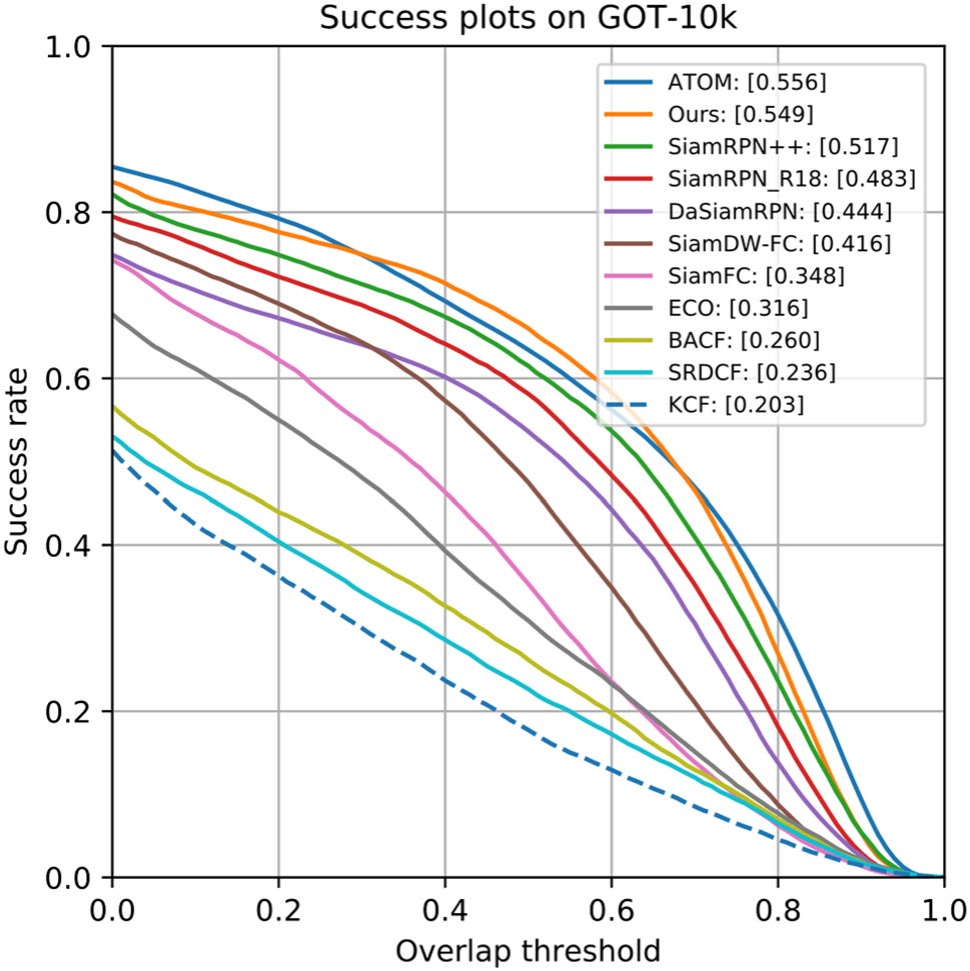
Success plots of our tracker and other 10 excellent methods on GOT-10k in regards to AO.

The GOT-10k consisting of 10k videos is a massive dataset. We make evaluation on GOT-10k test set with 180 videos. The GOT-10k test dataset has three indicators, including success plots, success rates (${SR}_{0.50}$ and ${SR}_{0.75}$) and average overlap (AO). In our experiment, we compare trackers according to ${SR}_{0.50}$, ${SR}_{0.75}$ and AO. The ${SR}_{i}$ represents the ratio of successfully tracked frames with overlap exceeds $i (i= 0.5, 0.75)$, while the AO represents the average overlaps between all predicting bounding boxes and ground-truth boxes. We follow the protocol of GOT-10k to make evaluation with our tracker and the other advanced trackers, that is, KCF^1^, SRDCF^3^, BACF^4^, ECO^12^, SiamFC^19^, SiamRPN^20^, DaSiamRPN^21^, SiamRPN++^25^, ATOM^56^. The evaluation results we used are obtained from the official GOT-10k website. As can be detailed seen in Table 2, our experimental results rank scores by 3.2%, 4.4%, 5.2% for AO, ${SR}_{0.50}$ and ${SR}_{0.75}$, respectively. Figure 7 shows that our tracker outperforms all those trackers on GOT-10k in terms of AO.

**Table 2:** Performance comparisons of our tracker with 9 excellent trackers on GOT-10k.

**Tracker**	**AO** $\uparrow$	${{\varvec{S}}{\varvec{R}}}_{0.50}\uparrow$	${{\varvec{S}}{\varvec{R}}}_{0.75}\uparrow$
KCF	0.203	0.177	0.065
SRDCF	0.236	0.227	0.094
BACF	0.260	0.262	0.101
SiamFC	0.348	0.353	0.098
ECO	0.316	0.309	0.111
SiamRPN_R18	0.483	0.581	0.270
DaSiamRPN	0.444	0.536	0.220
ATOM	**0.556**	*0.634*	**0.402**
SiamRPN++	***0.517***	***0.616***	***0.325***
Ours	*0.549*	**0.660**	*0.377*

Bold, Italic and bold-italic fonts represent the top-3 trackers on each indicator. $\uparrow$ denotes the highest is the best, and $\downarrow$ denotes the lowest is the best implementation.

### Ablation study

#### On network structure

To validate the performance of our tracker, we make the ablation study for our model on the VOT2016^37^ dataset. The verification results are listed in is reported in Table 3, We take SiamRPN^20^ as baseline, anchor-free framework and multiscale spatial attention extraction block are gradually added. The basic description is as follows. (a) ‘Baseline’ is the classical SiamRPN. (b) ‘Baseline + AF’ defines the baseline with an anchor-free framework. (c) ‘Baseline + NL’ is a tracker that we add non-local block to the baseline tracker. (d) ‘Baseline + AF + NL’ is a tracker that we add non-local block to the (b) tracker. (e) ‘Baseline + AF + SAE’ is our final model, which combines the baseline method with anchor-free framework and our proposed multiscale spatial attention extraction module. As we can see, our contribution improves the baseline by 4%, 14%, 11.1% in accuracy, robust and expected average overlap, respectively.

**Table 3:** Effects of each component in our method. Results are reported on VOT2016.

Method	EAO $\uparrow$	A $\uparrow$	R $\downarrow$
Baseline	0.337	0.578	0.312
Baseline + AF	0.371	0.608	0.261
Baseline + NL	0.418	0.608	0.224
Baseline + AF + NL	0.420	0.619	0.210
Baseline + AF + SAE	0.448	0.618	0.172

$\uparrow$ denotes the highest is the best, and $\downarrow$ denotes the lowest is the best.

#### On training data

In our experiment, we discuss the impact of different training datasets on our tracker. We train our model with COCO^30^, ImageNet-VID^31^, ImageNet-DET^31^ and YouTube-VOS^33^ at the first time, and achieve success of 0.626 and precision of 0.846. We then additionally add YouTube-BoundingBoxes^32^, and improve the performance by 1.7% and 1.2%. At last, we add GOT-10k^34^ to the above training sets, and achieve our current tracking results. The evolution results on OTB100 dataset are shown in Table 4. We can conclude from Table 4 that using the current large-scale training sets like YouTube-BoundingBoxes and GOT-10k for training can improve our tracking performance with 3.4% success and 4% precision on OTB100, while our model can still achieve the excellent performance using different choices of the tracking datasets.

**Table 4:** Results on OTB100 with different training datasets as listed.

Method	Training set	$\mathrm{Success}\uparrow$	$\mathrm{Precision}\downarrow$
Ours	VID, VOS, COCO, DET	0.639	0.860
Ours	VID, VOS, BB, COCO, DET	0.656	0.872
Ours	VID, VOS, BB, COCO, DET, GOT	0.673	0.900

$\uparrow$ denotes the highest is the best, and $\downarrow$ denotes the lowest is the best.

### Qualitative comparison

**Figure 8:**
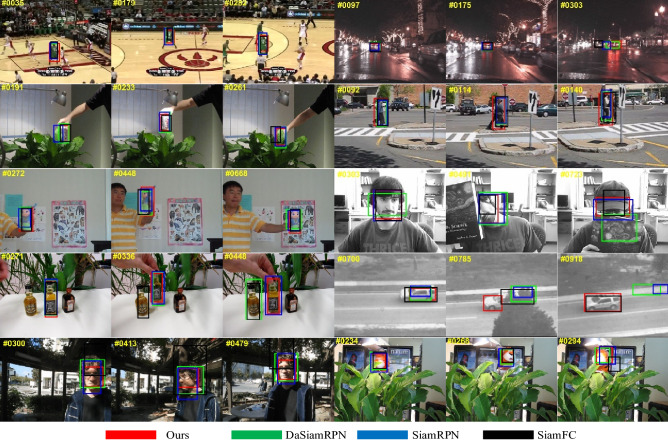
Qualitative comparison with three classical Siamese-based trackers on 8 challenging tracking scenarios of OTB100.

We select eight challenging tracking scenarios from OTB100 in this section. As shown in Fig. 8, from top to bottom, those tracking scenarios are basketball, carDark, coke, couple, doll, faceocc, liquor, suv, trellis, tiger. Due to our flexible anchor-free framework, the bounding boxes of our tracker can vary along with the change of the target during tracking phase. Compared to several classical FC-based and RPN-based trackers^19–21^, the proposed SAE block can capture considerable information around the target. In basketball, carDark and walking scenarios, those trackers such as SiamFC and SiamRPN cannot keep tracking the target in the following video frames. But due to the proposed SAE block, we can still pinpoint the target.

### Attributes comparison with excellent trackers

**Figure 9:**
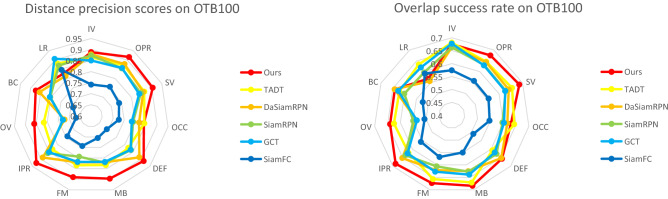
Precision and success plots of our tracker and those 5 excellent CNN-based trackers on the 11 challenges of OTB100.

**Figure 10:**
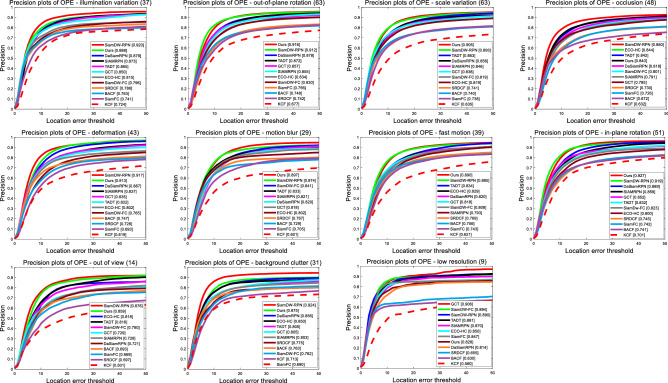
Detailed precision plots of our tracker and other 11 excellent trackers on the 11 challenges of OTB100.

**Figure 11:**
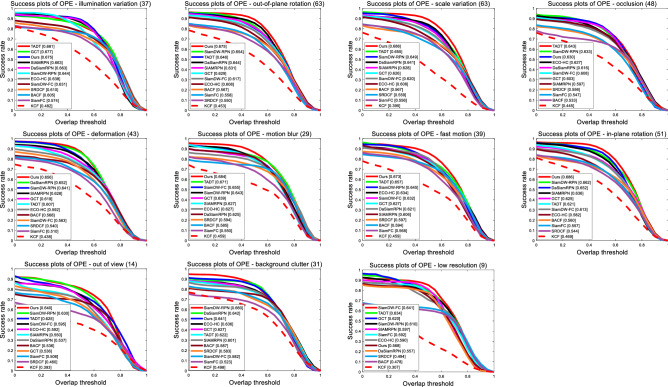
Detailed success plots of our tracker and other 11 excellent trackers on the 11 challenges of OTB100.

To evaluate the performance of our proposed tracker in dealing with many difficult challenges, we compare our algorithm with those advanced trackers using the 11 challenging object tracking scenarios of OTB100^35^ in detail, including out-of-plane rotation (OPR), in-plane rotation (IPR), deformation (DEF), occlusion (OCC), scale variation (SV), out of view (OV), fast motion (FM), motion blur (MB), background clutter (BC), low resolution (LR), illumination variation (IV). In Fig. 9, we compare our tracker with those advanced CNN-based trackers. We can conclude that our tracker is the most robust and accurate than other CNN-based trackers in most of aspects, such as out of view, fast motion, motion blur and scale variation, etc. In Figs. 10 and 11, we compare our trackers with other excellent trackers on those 11 challenging scenarios of OTB100 in detail. As we can see that our tracker performs top-3 in most of complex tracking scenarios. However, because of the proposed SAE block, we need to calculate more in each pixel that makes our tacker is not robust to track object in low resolution (LR) scenario than other advanced trackers slightly.

## Conclusion

In this paper, we put forward a high-performance object tracking framework, and train the deep Siamese model with an end-to-end fashion. Our proposed tracker directly predicts the label on each pixel of the search region and regress the prediction bounding boxes without requiring a multi-scale test or the pre-defined anchor boxes. Furthermore, we extract multiscale features through ResNet-50, and modify those features by the proposed spatial attention extraction block to enhance the ability of our model to obtain long-range dependencies. To demonstrate the generalizability of our tracker, we experiment our tracker on four mainstream challenging tracking benchmarks: OTB100, UAV123, VOT2016 and GOT-10k, and get the excellent results. Although our tracker can achieve considerable performance, it still cannot deal with challenges from low-resolution scenarios very well.
